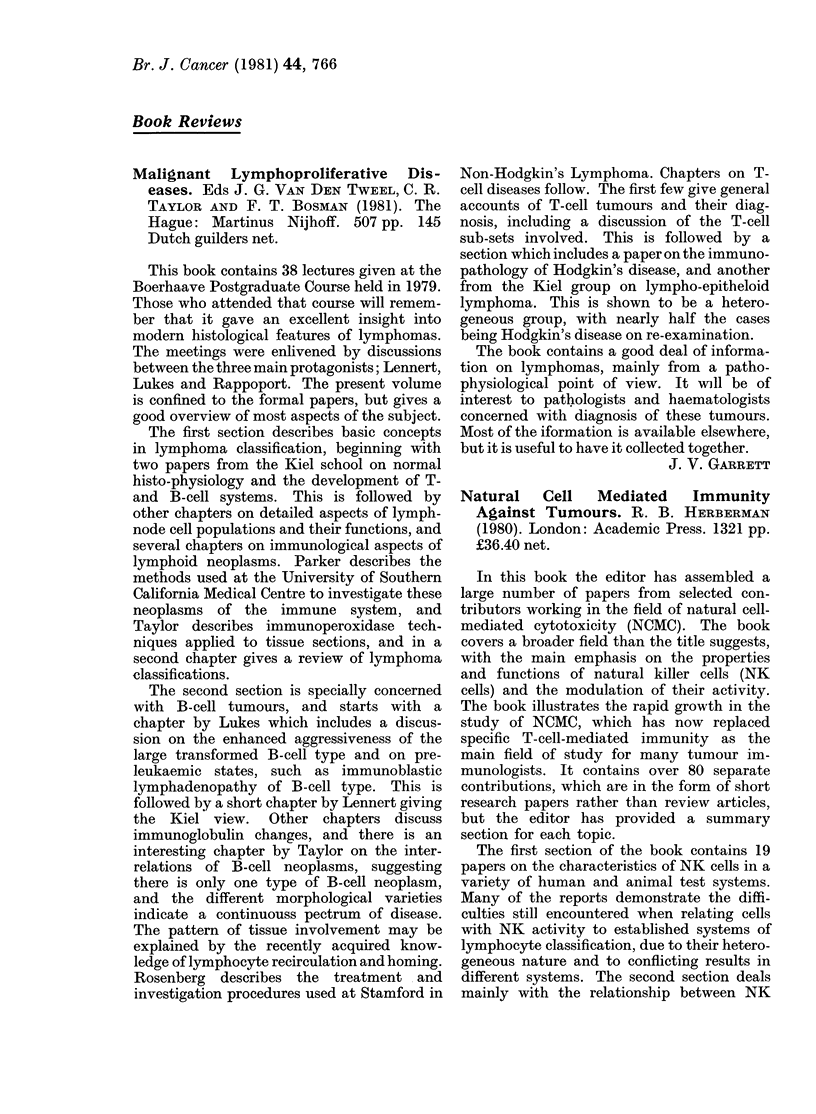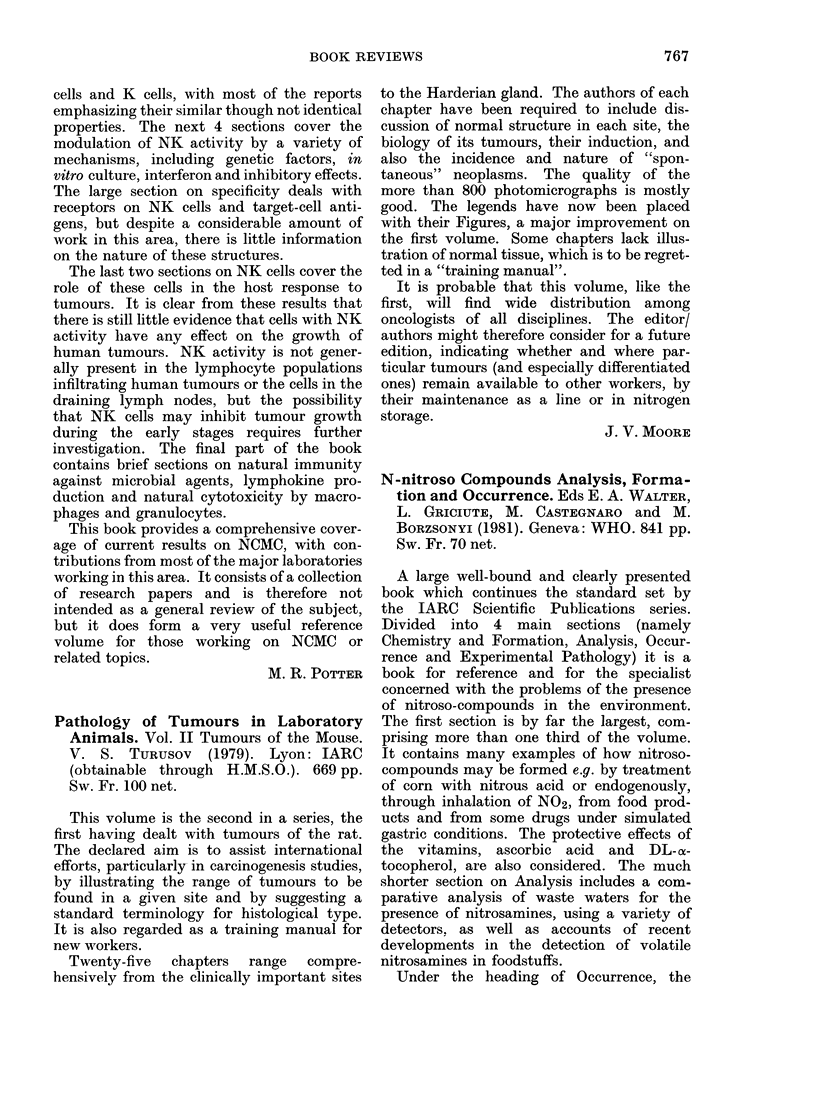# Natural Cell Mediated Immunity Against Tumours

**Published:** 1981-11

**Authors:** M. R. Potter


**Natural Cell Mediated Immunity Against Tumours.** R. B. Herberman (1980). London: Academic Press. 1321 pp. £36.40 net.

In this book the editor has assembled a large number of papers from selected contributors working in the field of natural cell-mediated cytotoxicity (NCMC). The book covers a broader field than the title suggests, with the main emphasis on the properties and functions of natural killer cells (NK cells) and the modulation of their activity. The book illustrates the rapid growth in the study of NCMC, which has now replaced specific T-cell-mediated immunity as the main field of study for many tumour immunologists. It contains over 80 separate contributions, which are in the form of short research papers rather than review articles, but the editor has provided a summary section for each topic.

The first section of the book contains 19 papers on the characteristics of NK cells in a variety of human and animal test systems. Many of the reports demonstrate the difficulties still encountered when relating cells with NK activity to established systems of lymphocyte classification, due to their heterogeneous nature and to conflicting results in different systems. The second section deals mainly with the relationship between NK

cells and K cells, with most of the reports emphasizing their similar though not identical properties. The next 4 sections cover the modulation of NK activity by a variety of mechanisms, including genetic factors, *in vitro* culture, interferon and inhibitory effects. The large section on specificity deals with receptors on NK cells and target-cell antigens, but despite a considerable amount of work in this area, there is little information on the nature of these structures.

The last two sections on NK cells cover the role of these cells in the host response to tumours. It is clear from these results that there is still little evidence that cells with NK activity have any effect on the growth of human tumours. NK activity is not generally present in the lymphocyte populations infiltrating human tumours or the cells in the draining lymph nodes, but the possibility that NK cells may inhibit tumour growth during the early stages requires further investigation. The final part of the book contains brief sections on natural immunity against microbial agents, lymphokine production and natural cytotoxicity by macrophages and granulocytes.

This book provides a comprehensive coverage of current results on NCMC, with contributions from most of the major laboratories working in this area. It consists of a collection of research papers and is therefore not intended as a general review of the subject, but it does form a very useful reference volume for those working on NCMC or related topics.

M. R. POTTER